# Readability, understandability and language accessibility of Swedish websites about the coronavirus disease 2019: a cross-sectional study

**DOI:** 10.1186/s12911-022-01873-y

**Published:** 2022-05-13

**Authors:** Susanne Georgsson, Tommy Carlsson

**Affiliations:** 1 The Swedish Red Cross University, Huddinge, Sweden; 2 Department of Clinical Science, Intervention and Technology, Karolinska Institutet, Stockholm, Sweden; 3 Department of Women’s and Children’s Health, Uppsala University, MTC-huset, Dag Hammarskjölds väg 14B, 1 tr, 75237 Uppsala, Sweden

**Keywords:** Consumer health information, COVID-19, Readability, Severe acute respiratory syndrome coronavirus 2, World wide web, Quality

## Abstract

**Background:**

The COVID-19 pandemic has caused significant morbidity and mortality. To mitigate its spread, members in the general population were prompted to apply significant behavioral changes. This required an effective dissemination of understandable information accessible for people with a wide range of literacy backgrounds. The aim of this study was to investigate the readability, understandability and language accessibility of Swedish consumer-oriented websites containing information about COVID-19.

**Methods:**

Websites were identified through systematic searches in Google.se (n = 76), and were collected in May 2020 when the pandemic spread started in Sweden. Readability and understandability were assessed with the Readability Index, the Ensuring Quality Information for Patients (EQIP) tool, and the Patient Education Materials Assessment Tool Understandability subscale (PEMAT-PU).

**Results:**

The median total sample score for Readability Index was 42.0, with the majority of scores being classified as moderate (n = 30, 39%) or difficult (n = 43, 57%). Median total sample scores were for EQIP 54.0% (IQR = 17.0, Range = 8–75) and for PEMAT-PU 60.0% (IQR = 14.75, Range = 12–87). The majority of the websites did not have any texts or links containing information in an alternative language (n = 58, 76%).

**Conclusions:**

Swedish websites contained information of difficult readability and understandability at the beginning of the coronavirus disease 2019 pandemic, with few providing information available in alternative languages. It is possible that these deficits contributed to the spread and impact of the virus. There is a need for studies investigating methods aiming to enhance the readability, understandability and language accessibility of web-based information at the beginning of an epidemic or pandemic.

**Supplementary Information:**

The online version contains supplementary material available at 10.1186/s12911-022-01873-y.

## Background

The Web is an enormous and highly popular resource for health-related information, highly utilized by a wide range of individuals within the general population [1, 2]. The Web has a great potential to empower consumers by offering high-quality information that is accessible and tailored according to their needs [3, 4]. It introduces an opportunity for people to access a great volume of health-related information when it is convenient for them, with the option to remain anonymous if they so desire [5]. When used correctly and appropriately, the Internet has the potential to improve health-related information uptake, enhance the patient-professional relationship, promote knowledge, and support decision-making processes [6]. All of these aspects are undoubtedly worth considering when implementing strategies to mitigate the spread of communicable diseases.

No universal mechanisms have been implemented to ensure high-quality websites providing information about health and wellbeing [3]. This illustrates a risk that online consumers encounter low-quality information that will fail to empower them towards informed decisions and healthy behaviors. For health-related information to be adequately understood and accurately interpreted, the recipient needs to have sufficient health literacy. Personal health literacy is defined as “the degree to which individuals have the ability to find, understand, and use information and services to inform health-related decisions and actions for themselves and others”, while organizational health literacy concerns the extent to which organizations enable these decisions and actions [7]. Low health literacy in the general population is an acknowledged challenge, with studies indicating high prevalence of low levels, and conversely, a low prevalence of proficient levels [8]. Repeatedly, low health literacy has been shown to be associated with increased hospitalization and emergency care use, and among older populations, worsened health status and higher mortality [9]. One way to address low health literacy is to disseminate content written in a plain, easy-to-read, clear, and accessible language [10].

In early 2020, the coronavirus COVID-19 caused a pandemic with health-related consequences across the globe. The pandemic caused significant morbidity and mortality in Europe, including Sweden [11]. As the disease spread, members of the general population were faced with unfamiliar challenges related to disease prevention in their daily life, which required health-related behavioral changes [12]. Thus, the public had a considerable demand for high-quality information about aspects such as disease prevention, symptoms and treatment [13]. Indeed, timely and adequate communication is essential in order to empower individuals to make autonomous and informed health-related decisions [14]. Considering the prevalent utilization of the Web as a resource for health-related information, some studies investigating the readability of such materials have been conducted. These empirical studies have utilized a range of various automated readability formulas [15–21], all concluding that web-based information about COVID-19, written in the English language, is difficult to read [15, 17, 21–23]. Previous results uniformly show that only a small minority of websites meet the recommended 6^th^ grade readability level [15, 16, 18–21]. However, little is known about the readability, understandability, and accessibility of websites containing information in non-English languages. There are a great number of languages represented in Europe, calling attention to the considerable challenge of disseminating high-quality information so that it reaches the diverse population. An important finding in one previous study was the lack of readily translated information and limited utilization of graphic-based material about COVID-19 [17], raising questions about the accessibility of translated information in non-English speaking settings. A further problem with the previously conducted studies is the general reliance on automated readability formulas, which do not capture intrinsic design elements and various multi-dimensional aspects related to the presentation of the web-based information, often referred to in the literature as understandability [24].

Taken together, there is a need to systematically assess the quality of the information disseminated at the beginning of the pandemic in non-English speaking settings, utilizing both automated readability tests as well as assessments of understandability. The overall aim of this study was to add to the current knowledge about the quality of web-based information about COVID-19 by providing a systematic assessment of information written in the Swedish language. Specifically, our objectives were to investigate: (1) the readability of information in the Swedish language through an automated readability formula; (2) the understandability by systematically assessing aspects of language and presentation not captured with an automated formula; and (3) determine the accessibility of readily translated versions of original web-based information written in the Swedish language.

## Methods

### Design

This was a descriptive cross-sectional study utilizing quantitative variables including automated readability calculations and systematic assessments based on established instruments. This study is an in-depth analysis of a data set previously analyzed in regard to quality variables other than those analyzed herein [25]. This study is reported according to the STROBE checklist for cross-sectional studies (Additional file 1).

### Data collection

In May 2020, a set of 17 searches were performed in the Swedish version of Google (Google.se), the most popular search engine in Sweden [26]. Based on previous research reporting search patterns in the general population, we used several search strings, screened the first 20 links in each hit list, and retrieved the information presented in the first web page of each link [27, 28]. The search strings were inspired by popular and rising COVID-19 related search terms in Google Trends. No quotation marks or other search engine operators were used. The search strings are presented in detail in Additional file 2. The terms involved common Swedish terms for COVID-19, the coronavirus, disease prevention, self-care, and symptoms. A total of 340 hits were screened for inclusion using the Web browser Google Chrome, set to incognito mode in order to limit the impact of previous searches. To be included, websites needed to contain text-based information about COVID-19 intended for the general population, be written in Swedish, and be accessible without any password or payment requirements. Websites providing information specifically developed for health professionals were excluded, based on the assumption that we aimed to investigate the quality of information intended for the general population.

In the initial screening, 97 websites were excluded because they were irrelevant (n = 74), not written in Swedish (n = 11), and were inaccessible (n = 12). Among the remaining websites, 35 were excluded because they were written for health professionals (n = 33) or did not contain any text-based content (n = 2). After correcting for duplicate hits (n = 132), 76 unique websites were included in the final sample. All included websites were captured with NCapture in May 2020, to save the content as it was published at the time of data collection.

### Data analysis

The data were analyzed with an automated readability formula, two tools for systematic assessment of understandability, and by recording readily available translated versions of the included websites. The last author, a specialist intensive care nurse and midwife who is a researcher and associate professor, performed all assessments. Additional file 3 presents the dataset of readability and understandability scores for the included websites.

#### Readability

The readability of the text-based content in all included websites were calculated with Readability Index (Swedish: Läsbarhetsindex, LIX), an automated formula used to determine the readability of Swedish texts. Scores less than 25 indicate easy readability, while scores over 60 indicate difficult readability. A score over 40 signals that the readability is too difficult for an average person to fully comprehend [29]. The corresponding grade levels of LIX scores are: less than 28 represent elementary school (grades 1–5), 28–43 represent junior high school (grades 6–9), 44–55 represent senior high school (grades 10–12) and more than 55 represent college or university [30].

#### Understandability

Understandability was assessed with The Ensuring Quality Information for Patients (EQIP) tool and the understandability subscale in the Patient Education Materials Assessment Tool for printable materials (PEMAT-PU). Both instruments have been shown to have adequate validity and reliability [31–33]. EQIP assesses a set of quality criteria related to language, visual aids, tone, and design/layout. Thirteen questions are rated as yes (1), partially (0.5), no (0), or not applicable [32]. To determine the overall score, the sum of the EQIP ratings are divided with the total number of applicable items and multiplied with 100, generating a percentage score between 0 and 100%. Higher scores indicate better quality. An EQIP score above 75% is considered high quality, 51–75% is considered good quality with minor problems, and scores below 51% is considered serious or severe problems in quality [32].

PEMAT-PU assesses a set of quality criteria related to content, word choice and style, use of numbers, organization, layout and design, and use of visual aids. Seventeen questions are rated as agree (1), disagree (0), or not applicable [33]. To determine the overall score, the sum of the ratings in the PEMAT-PU are divided with the total number of applicable items and multiplied with 100, generating a percentage score between 0 and 100%. Higher scores indicate better quality. A PEMAT-PU score of 70% has been suggested as a cut-off value, indicating that websites scoring less than 71% would be considered poorly understandable [33].

#### Language accessibility

All websites were thoroughly read through repeatedly and all translated sections or links to translations in an alternative language found within were recorded. Links leading to automated translations utilizing external services such as Google Translate were not considered readily available translations.

## Results

### Website affiliations

Within the total sample of 76 unique included websites, they were affiliated with the government (n = 19, 25%), health care services (n = 17, 22%), newspapers (n = 17, 22%), information websites produced by independent companies with the sole purpose to provide web-based information (n = 9, 12%), pharmacies (n = 5, 7%), and nine websites (12%) were categorized as having other affiliation (humanitarian organizations, n = 2; universities, n = 2; insurance company, n = 1; medical products company, n = 1; online health food store, n = 1; patient organization, n = 1; wiki page, n = 1).

### Readability

The median score for Readability Index (LIX) was 42.0 for the total sample (Table 1), with the majority of the readability scores being classified as moderate (n = 30, 39%) or difficult (n = 43, 57%), Table 2. The highest median LIX scores, illustrating the most difficult readability within the sample, were found for government-affiliated websites (Med = 44.0, IQR = 8.5). The lowest median LIX scores, illustrating the easiest readability within the sample, were found for websites affiliated with health care (Med = 39.0, IQR = 6.0). However, none of the included websites in the total sample were classified as having very easy readability, and only one website affiliated with news was classified as having easy readability. As depicted in Additional file 4, the lowest LIX scores, indicating the easiest readability within the sample, were found in websites affiliated with news (LIX = 29 and 33) and health care (LIX = 31). Conversely, the highest LIX scores, indicating most difficult readability within the sample, were found in websites affiliated with the government (LIX = 50, 53, and 54).

**Table 1:** Automated readability scores and assessment scores for included websites (n = 76)

Instrument	Affiliation	Median (IQR)	Quality	Range
Readability Index (LIX)	Government	44.0 (8.5)	Difficult	35–54
	Health care	39.0 (6.0)	Moderate	31–49
	News	41.0 (7.0)	Difficult	29–47
	Information website	43.0 (5.0)	Difficult	35–48
	Pharmacy	41.0 (2.0)	Difficult	38–47
	Other affiliation	43.0 (4.0)	Difficult	35–47
	Total sample	42.0 (6.25)	Difficult	29–54
EQIP	Government	54.0 (17.0)	Good quality with minor problems	8–73
	Health care	54.0 (19.0)	Good quality with minor problems	38–75
	News	58.0 (20.0)	Good quality with minor problems	35–75
	Information website	50.0 (16.0)	Serious or severe problems in quality	19–58
	Pharmacy	42.0 (12.0)	Serious or severe problems in quality	38–58
	Other affiliation	50.0 (12.0)	Serious or severe problems in quality	38–58
	Total sample	54.0 (17.0)	Good quality with minor problems	8–75
PEMAT-P Understandability	Government	67.0 (9.0)	Poorly understandable	17–87
	Health care	60.0 (14.0)	Poorly understandable	42–67
	News	56.0 (10.0)	Poorly understandable	31–78
	Information website	47.0 (13.0)	Poorly understandable	12–63
	Pharmacy	67.0 (14.0)	Poorly understandable	40–80
	Other affiliation	60.0 (2.0)	Poorly understandable	47–67
	Total sample	60.0 (14.75)	Poorly understandable	12–87

**Table 2:** Readability levels for the included websites (n = 76)

Score	Level	Government	Health care	News	Information website	Pharmacy	Other	Total
< 25	Very easy	–	–	–	–	–	–	–
25–30	Easy	–	–	1	–	–	–	1
31–40	Moderate	6	9	7	3	2	3	30
41–50	Difficult	11	8	9	6	3	6	43
51–60	Very difficult	2	–	–	–	–	–	2
> 60	Most difficult	–	–	–	–	–	–	–

### Understandability

**Fig. 1:**
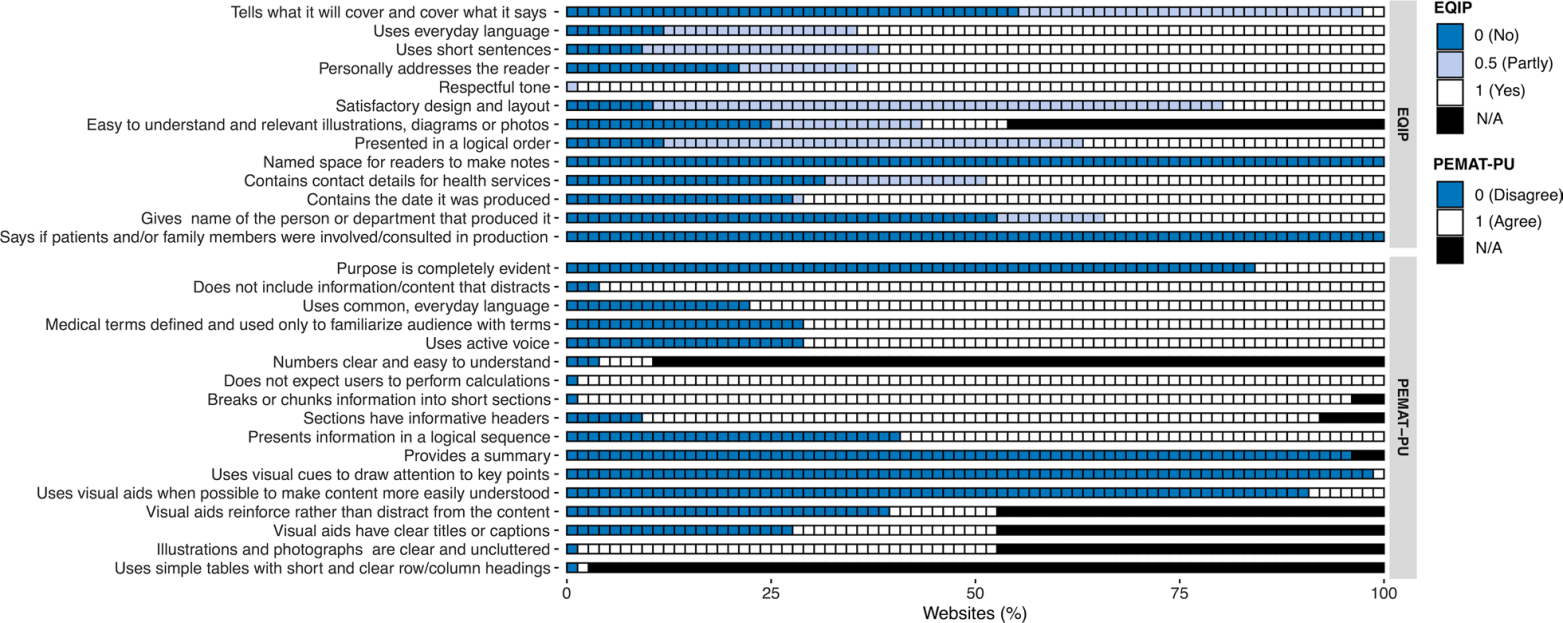
Distributions of understandability assessment scores for the included websites (n = 76)

The median EQIP score was 54.0% (IQR = 17.0, Range = 8–75), approaching a score classified as serious or severe problems in quality (Table 1). In total, seven questions in EQIP had more than half of the included websites not or only partly adhering to the criteria (Fig. 1). While all median EQIP scores indicated quality deficits, sources affiliated with information websites (Med = 50.0, IQR = 16.0) and pharmacies (Med = 42.0, IQR = 12.0) had median scores below 51%, indicating serious or severe problems. As depicted in Additional file 4, the highest EQIP scores, indicating highest quality, were found in websites affiliated with news (75%), health care (75%), and the government (73%). Conversely, the lowest EQIP scores, indicating low quality, were found in websites affiliated with the government (8% and 29%) and an information website (19%).

The median PEMAT-PU score was 60.0% (IQR = 14.75, Range = 12–87), indicating poorly understandable content (Table 1). In total, four of the questions in PEMAT-PU had more than half of the websites not adhering to the criteria (Fig. 1). Regardless of website affiliation, all median PEMAT-PU scores indicated poorly understandable information, with lowest median PEMAT-PU scores among sources affiliated with newspapers (Med = 56.0%, IQR = 10.0) and information websites (Med = 47.0%, IQR = 13.0). As depicted in Additional file 4, the highest PEMAT-PU scores, indicating the most understandable content, were found in websites affiliated with the government (87% and 80%) and a pharmacy (80%). Conversely, the lowest PEMAT-PU scores, indicating least understandable content, were found in websites affiliated with news (31%), the government (17%), and an information website (12%).

### Language accessibility

The most common translated sections/links to information in an alternative language in the included websites were English (n = 12, 16%), sign language (n = 8, 11%), and Arabic (n = 6, 8%). The least common were Farsi (n = 1, 1%), Romani kelderash (n = 1, 1%), and Syrian (n = 1, 1%), Table 3. The median number of websites with links to information in alternative languages was 0 (Range 0–30), with the majority of the websites not containing any link to information in an alternative language other than Swedish (n = 58, 76%). Eleven (14%) websites contained links to one alternative language, two (3%) contained links to two alternative languages, one (1%) contained links to three alternative languages, and four (5%) contained links to more than six alternative languages.

**Table 3:** Websites with links to information in other languages

Language	n (%)
English	12 (16%)
Sign language	8 (11%)
Arabic	6 (8%)
Finnish	5 (7%)
Somali	5 (7%)
Dari	4 (5%)
Tigrinya	4 (5%)
Amharic	3 (4%)
French	3 (4%)
German	3 (4%)
Kurmanji	3 (4%)
Lule sami	3 (4%)
Meänkieli	3 (4%)
Northern sami	3 (4%)
Pashto	3 (4%)
Persian	3 (4%)
Polish	3 (4%)
Romani arli	3 (4%)
Romani lovari	3 (4%)
Russian	3 (4%)
Sorani	3 (4%)
Sothern sami	3 (4%)
Spanish	3 (4%)
Thai	3 (4%)
Turkish	3 (4%)
Bosnian	2 (3%)
Chinese	2 (3%)
Croatian	2 (3%)
Serbian	2 (3%)
Farsi	1 (1%)
Romani kelderash	1 (1%)
Syrian	1 (1%)

## Discussion

The aim of this study was to investigate the readability, understandability and language accessibility of consumer-oriented websites about COVID-19 written in the Swedish language, revealing moderate to difficult readability levels, poor understandability, and limited language accessibility. The COVID-19 pandemic has illustrated the importance of strategies implemented to ensure the dissemination of high-quality information about disease prevention. To the extent of our knowledge, this is the first study to investigate the readability of Swedish consumer-oriented websites about COVID-19 at the beginning of the pandemic. The findings resonate what has been reported regarding difficult readability determined with automated readability calculations for websites written in the English language [15, 17, 21–23]. Established recommendations state that web-based sources should be written on a sixth-grade reading level [34], and our findings as well as other studies investigating readability in other languages confirm that the readability of web-based information about COVID-19 far exceeds this goal [15, 17, 21–23]. The median LIX score was 42, corresponding to approximately 9th to 10th grade readability level. Similar grade levels (ranging from 8.7 to 14.3) have been reported in previous studies investigating websites written in the English language, confirming our findings and giving further weight to the problematic situation on the Web [15, 17, 18, 20, 21]. In our study, difficult readability was found regardless of website affiliation, including websites originating from sources patients traditionally rely on, such as health care services and the government. Interestingly, one previous study observed easier readability of sources affiliated with the government and public health services, albeit still more difficult than the recommended readability grade level [21]. Other studies, on the other hand, report very small or insignificant differences between website affiliations [15, 17, 20]. This calls attention to the need for measures aiming to enhance the readability and understandability of online sources in general.

Understandability is acknowledged in the literature as a core quality criteria for web-based information, capturing nuances of presentation, writing, and language not represented within automated readability formulas [24]. As of yet, understandability of web-based sources about COVID-19 has not been extensively investigated in research. One study indicate good understandability with a PEMAT score of 83% [18], illustrating results conflicting with our findings and a need for further investigation in order to draw firm conclusions. Research has shown that complementing standard information routes with non-textual media, such as relevant and clear illustrations, has the potential to increase information uptake and combat low health literacy [35]. However, only 7 (9%) of the included websites in our study utilized visual aids when possible to make content more easily understood. Another study report very similar findings, with 7% of the websites therein providing graphical information [17]. Taken together, the results of our study highlights a high probability that members in the general population are faced with texts containing information of difficult readability and low understandability when they turn to the Web for information about COVID-19. We acknowledge an urgent need to improve quality standards on the Web and highly encourage future research that addresses this challenge.

The COVID-19 pandemic placed substantial challenges and responsibilities on all persons within the general population, involving health-related behavioral changes related to preventive measures applied in their daily lives [36]. As a response to these unfamiliar and crucial circumstances, a high demand on high-quality and continuously updated information was seen [13, 37]. High COVID-19 hospitalization and mortality has been reported in Sweden, which has been shown to be associated with the proportion of migrants living in certain geographical areas [38]. Moreover, high risk to contract COVID-19 when not speaking the native language in a country has been reported [39], calling further attention to the need for reliable information available in a range of different languages. A review of government produced risk communication about COVID-19 revealed that a considerable proportion of countries in Europe lack translated information, concluding substantial and important gaps in the availability translated information available for non-native speakers [40]. According to another study investigating online information in the UK, there has been a lack of resources and appropriate COVID-19 online educational material available to minority groups, with substandard readability and a significant lack of translated information [17]. Our results give further weight to this problem, showing that the majority of the included websites only included Swedish information. Sweden has a diverse population, with many ethnic backgrounds represented and a significant proportion not being native speakers. Our findings indicate a high possibility that non-Swedish speaking persons seeking high-quality information about COVID-19 grounded in a Swedish context experienced difficulties finding online information written in an alternative language.

Understanding information about preventive measures, including when and how to apply these in daily life, is necessary in order to mitigate the spread of infectious diseases leading to epidemics and pandemics [12, 41]. The difficult readability, poor understandability and lack of readily translated material could have affected their comprehension and retention of the information, which may have affected the spread of the disease, and this should be addressed in future studies. We acknowledge that some persons may use automated translation services such as Google translate when accessing information about COVID-19. We investigated readily available translated versions of Swedish information and cannot make any conclusions about the reliability of using these automated services. Our findings call attention to the need for systematic approaches among developers of consumer-oriented health-related information on the Web to provide information that is both readable and understandable. Utilizing easy-to-understand language translated in alternative languages and accompanied by appropriate visual aids has the potential to enhance knowledge within the general population, but more research about this is needed.

### Methodological considerations

There are methodological limitations that need to be considered when interpreting the results of this study. The searches were designed with the intent to mimic search patterns in the general population, but we cannot dismiss the risk of not identifying some online sources used by the general population. Information consumers may utilize search terms in other languages, other search engines, or other language versions of Google. This would affect which websites that would be accessed and would thus affect the generalizability. Our findings need to be interpreted together with other studies investigating websites in other languages and settings. Readability was determined with Readability Index (LIX), which is a popular and established method used to produce a quantified score based on the number of words in sentences and the proportion of long words [29]. Automated readability formulas have been criticized for producing a simplified understanding of readability determined through formal text properties, not taking into account other complex aspects such as word familiarity and medical jargon [42]. Therefore, we also assessed each included website with two systematic instruments focusing on interaction aspects such as language, understandability, visual aids, tone, and presentation. Readability and literacy are complex concepts that are not easily captured in full. We acknowledge that the utilized formulas and systematic tools in our study have intrinsic limitations and do not take into account the health literacy of the recipient. Thus, we encourage further studies within this field of research.

The assessments confirmed substandard quality, performed by a researcher and associate professor. We acknowledge a need to conduct research that utilize laypersons as assessors of website quality, particularly those with low health literacy and reading texts in their non-native language. One assessor, who is a health professional and a researcher, scored the included websites. While other studies have shown adequate interrater reliability when utilizing EQIP [31] and PEMAT [43], this is nevertheless a limitation that needs to be considered when interpreting the findings. The large majority of studies investigating readability of online information about COVID-19 have been based on English content, and therefore, we argue that our study brings novel and important findings. Nevertheless, the generalizability of the results needs to be taken into consideration when transferring it to other contexts.

## Conclusion

According to our findings, Swedish websites about the coronavirus disease 2019 contain information of difficult readability and poor understandability. Links to information available in alternative languages was very scarce. It is possible that these deficits contributed to the spread and impact of the virus. We encourage more studies investigating methods to increase the readability, understandability and language accessibility of web-based information at the beginning of an epidemic or pandemic.

## Supplementary Information

**Additional file 1**. STROBE checklist for cross-sectional studies.

**Additional file 2**. Searches in Google.se.

**Additional file 3**. Data set.

**Additional file 4**. Quality scores of the top and bottom three websites in regard to the investigated variables.

## Data Availability

All data generated or analysed during this study are included in this published article and its supplementary information files.

## Abbreviations

COVID-19: The Coronavirus Disease 2019
LIX: Readability Index [Läsbarhetsindex]
EQIP: The Ensuring Quality Information for Patients tool
PEMAT-PU: The Patient Education Materials Assessment Tool for printable
