# Supplementary material for: Readability, understandability and language accessibility of Swedish websites about the coronavirus disease 2019: a cross-sectional study
Source: BMC Med Inform Decis Mak. 2022 May 13;22:131. doi: 10.1186/s12911-022-01873-y (PMC9103604; doi:10.1186/s12911-022-01873-y)
Supplement: Supplementary file 1 — Additional file 1. STROBE checklist for cross-sectional studies. [file 12911_2022_1873_MOESM1_ESM.pdf]

Additional File 1. STROBE Statement—Checklist of items that should be included in reports of cross-sectional studies.

	Item No	Recommendation	Found in manuscript
**Title and abstract**	1	(*a*) Indicate the study's design with a commonly used term in the title or the abstract	Title
		(*b*) Provide in the abstract an informative and balanced summary of what was done and what was found	Abstract
**Introduction**			
Background/rationale	2	Explain the scientific background and rationale for the investigation being reported	Background paragraphs 1 - 4
Objectives	3	State specific objectives, including any prespecified hypotheses	Background, paragraph 4
**Methods**			
Study design	4	Present key elements of study design early in the paper	Methods, Design Paragraph 1
Setting	5	Describe the setting, locations, and relevant dates, including periods of recruitment, exposure, follow-up, and data collection	Methods, Data Collection Paragraph 1
Participants	6	(*a*) Give the eligibility criteria, and the sources and methods of selection of participants	Methods, Data Collection Paragraph 1
Variables	7	Clearly define all outcomes, exposures, predictors, potential confounders, and effect modifiers. Give diagnostic criteria, if applicable	Methods, Data Analysis Paragraphs 1 - 5
Data sources/ measurement	8*	For each variable of interest, give sources of data and details of methods of assessment (measurement). Describe comparability of assessment methods if there is more than one group	Methods, Data Analysis Paragraph 1 - 5
Bias	9	Describe any efforts to address potential sources of bias	N/A
Study size	10	Explain how the study size was arrived at	Methods, Data Collection Paragraph 2
Quantitative variables	11	Explain how quantitative variables were handled in the analyses. If applicable, describe which groupings were chosen and why	Methods, Data Analysis Paragraph 1 - 5
Statistical methods	12	(*a*) Describe all statistical methods, including those used to control for confounding	Methods, Data Analysis Paragraph 1 - 5
		(*b*) Describe any methods used to examine subgroups and interactions	N/A
		(*c*) Explain how missing data were addressed	N/A
		(*d*) If applicable, describe analytical methods	N/A

		taking account of sampling strategy	
		(*e*) Describe any sensitivity analyses	N/A
**Results**			
Participants	13*	(a) Report numbers of individuals at each stage of study—eg numbers potentially eligible, examined for eligibility, confirmed eligible, included in the study, completing follow-up, and analysed	Methods, Data Collection Paragraph 2
		(b) Give reasons for non-participation at each stage	Methods, Data collection Paragraph 2
		(c) Consider use of a flow diagram	N/A
Descriptive data	14*	(a) Give characteristics of study participants (eg demographic, clinical, social) and information on exposures and potential confounders	Results, Website characteristics Paragraph 1
		(b) Indicate number of participants with missing data for each variable of interest	N/A
Outcome data	15*	Report numbers of outcome events or summary measures	Results, Readability and Understandability Paragraph 1; Language Accessibility Paragraph 1
Main results	16	(*a*) Give unadjusted estimates and, if applicable, confounder-adjusted estimates and their precision (eg, 95% confidence interval). Make clear which confounders were adjusted for and why they were included	Results, Readability and Understandability Paragraph 1; Language Accessibility Paragraph 1
		(*b*) Report category boundaries when continuous variables were categorized	Methods, Data analysis Paragraph 1; Results, Readability Paragraph 1; Results Understandability Paragraph 1; Results Language Accessibility Paragraph 1
		(*c*) If relevant, consider translating estimates of relative risk into absolute risk for a meaningful time period	N/A
Other analyses	17	Report other analyses done—eg analyses of subgroups and interactions, and sensitivity analyses	N/A
**Discussion**			
Key results	18	Summarise key results with reference to study objectives	Discussion, Principal Results Paragraph 1
Limitations	19	Discuss limitations of the study, taking into account sources of potential bias or imprecision. Discuss both direction and magnitude of any potential bias	Discussion, Methodological Considerations Paragraph 1
Interpretation	20	Give a cautious overall interpretation of results considering objectives, limitations, multiplicity of analyses, results from similar studies, and other relevant evidence	Discussion, Principal Results Paragraphs 1 - 4

Generalisability	21	Discuss the generalisability (external validity) of the study results	Discussion, Methodological Considerations Paragraph 1
**Other information**			
Funding	22	Give the source of funding and the role of the funders for the present study and, if applicable, for the original study on which the present article is based	Declarations, Funding Paragraph 1

*Give information separately for exposed and unexposed groups.

Note: An Explanation and Elaboration article discusses each checklist item and gives methodological background and published examples of transparent reporting. The STROBE checklist is best used in conjunction with this article (freely available on the Web sites of PLoS Medicine at http://www.plosmedicine.org/, Annals of Internal Medicine at http://www.annals.org/, and Epidemiology at http://www.epidem.com/). Information on the STROBE Initiative is available at www.strobe-statement.org.
