# Supplementary material for: Readability, understandability and language accessibility of Swedish websites about the coronavirus disease 2019: a cross-sectional study
Source: BMC Med Inform Decis Mak. 2022 May 13;22:131. doi: 10.1186/s12911-022-01873-y (PMC9103604; doi:10.1186/s12911-022-01873-y)
Supplement: Supplementary file 2 — Additional file 2. Searches in Google.se. [file 12911_2022_1873_MOESM2_ESM.pdf]

Additional File 2. Searches in Google.se

Search string [Swedish]	Search string [translated]	Included hits	
		Unique	Duplicate
Coronavirus förebygga	Coronavirus prevention	12	0
Covid förebygga	Covid prevention	1	4
Covid-19	Covid-19	3	1
Hur undviker jag corona	How do I avoid corona	10	8
Hur undviker jag covid-1913	How do I avoid covid-19	7	10
Hur förhindrar jag att smittas av corona	How do I prevent being infected by corona	5	11
Hur förhindrar jag att smittas av covid-19	How do I prevent being infected by covid-19	1	14
Hur skyddar jag mig från corona	How do I protect myself from corona	6	13
Hur skyddar jag mig från covid-19	How do I protect myself from covid-19	3	16
Egenvård corona	Self care corona	8	4
Egenvård covid-19	Self care covid-19	2	11
Hur lindrar jag coronainfektion	How do I alleviate corona infection	8	7
Hur lindrar jag covid-19	How do I alleviate covid-19	2	11
SARS-CoV-2	SARS-CoV-2	1	1
Virus corona	Virus corona	3	6
Coronavirus tips	Coronavirus tips	3	4
Coronavirus symtom	Coronavirus symptoms	1	11
Total		76	132
