# Supplementary material for: Readability, understandability and language accessibility of Swedish websites about the coronavirus disease 2019: a cross-sectional study
Source: BMC Med Inform Decis Mak. 2022 May 13;22:131. doi: 10.1186/s12911-022-01873-y (PMC9103604; doi:10.1186/s12911-022-01873-y)
Supplement: Supplementary file 3 — Additional file 3. Data set. [file 12911_2022_1873_MOESM3_ESM.pdf]

PEMAT1	PEMAT2	PEMAT3	PEMAT4	PEMAT5	PEMAT6	PEMAT7	PEMAT8	PEMAT9	PEMAT10	PEMAT11	PEMAT12	PEMAT13	PEMAT14	PEMAT15	PEMAT16
0	1	1	1	1	NA	1	1	1	1	0	0	0	NA	NA	NA
0	1	0	0	0	NA	1	1	1	1	0	0	0	0	1	1
1	1	1	1	0	NA	1	1	1	1	0	0	0	NA	NA	NA
0	1	1	1	1	NA	1	1	1	1	0	0	0	0	0	1
0	1	1	1	1	NA	1	1	1	1	0	0	0	0	0	1
0	1	0	0	0	NA	1	1	1	1	0	0	1	1	1	1
0	0	1	0	1	NA	1	1	1	0	0	0	0	NA	NA	NA
1	1	1	1	1	NA	1	NA	NA	1	NA	0	0	NA	NA	NA
0	1	1	1	1	NA	1	1	1	1	0	0	1	1	1	1
0	1	1	0	1	1	1	1	1	1	0	0	1	1	1	1
1	1	1	1	1	NA	1	1	1	1	0	0	0	NA	NA	NA
0	1	1	1	1	NA	1	1	0	1	0	0	0	NA	NA	NA
0	1	0	0	0	NA	1	1	1	1	0	0	0	0	1	1
0	1	1	1	1	NA	1	1	1	1	0	0	0	NA	NA	NA
0	1	1	0	1	NA	1	1	1	1	0	0	0	NA	NA	NA
0	1	0	0	0	NA	1	1	1	1	0	0	1	1	1	1
1	1	1	1	1	NA	1	1	1	1	0	0	0	NA	NA	NA
1	1	1	1	1	NA	1	1	1	0	0	0	0	0	0	1
0	1	1	1	1	1	1	1	1	1	0	0	0	0	0	1
0	1	1	1	0	NA	1	1	1	1	0	0	0	NA	NA	NA
1	1	1	1	1	1	1	1	1	0	0	0	0	0	1	1
0	1	1	0	0	1	1	1	1	1	0	0	0	1	0	1
0	1	1	1	1	NA	1	1	0	0	0	0	0	NA	NA	NA
0	1	1	0	0	NA	1	1	1	0	0	0	0	0	0	1
1	1	1	1	0	NA	1	1	1	0	0	0	0	0	1	1
1	1	1	1	0	NA	1	1	1	1	0	0	0	0	1	1
1	1	1	1	1	NA	1	1	1	1	0	0	1	1	0	1
0	1	1	1	1	NA	1	1	1	0	0	0	0	NA	NA	NA
0	1	1	1	1	NA	1	NA	NA	1	NA	0	0	NA	NA	NA
0	1	1	1	1	NA	1	1	1	1	0	0	0	1	0	1
0	1	1	1	1	NA	1	0	0	0	0	0	0	1	1	1
0	1	0	0	1	NA	1	1	1	0	0	0	0	0	0	1
1	1	0	1	1	1	1	1	1	1	0	0	0	0	0	1
0	1	1	1	1	NA	1	1	1	1	0	0	0	NA	NA	NA
0	1	1	1	0	NA	1	1	0	1	0	0	0	NA	NA	NA
0	1	0	1	0	NA	1	1	1	1	0	0	0	0	1	1
0	1	1	1	1	NA	1	1	1	1	0	0	0	NA	NA	NA
0	1	1	1	1	NA	1	1	1	1	0	0	0	NA	NA	NA
0	1	1	1	1	NA	1	1	1	0	0	0	0	NA	NA	NA
0	1	1	1	1	NA	1	1	1	1	0	0	0	0	0	1
0	1	0	0	0	0	1	1	1	1	0	0	1	1	1	1
0	1	1	1	0	NA	1	1	1	0	0	0	0	NA	NA	NA
0	1	0	0	0	0	1	1	1	0	0	0	0	0	0	1
0	0	0	0	0	0	0	1	1	0	0	0	0	0	0	0
0	1	1	1	1	NA	1	1	1	1	0	0	0	0	0	1
0	1	1	1	1	NA	1	1	NA	1	NA	0	0	NA	NA	NA
0	1	1	1	1	NA	1	1	1	0	0	0	0	0	0	1
0	1	1	1	1	NA	1	1	1	1	0	0	0	NA	NA	NA
0	1	1	1	1	NA	1	1	1	0	0	0	0	NA	NA	NA
0	1	1	0	1	NA	1	1	0	0	0	0	0	0	1	1
0	1	0	0	1	NA	1	1	1	0	0	0	0	NA	NA	NA
0	1	0	0	1	NA	1	1	1	0	0	0	0	0	1	1
0	1	1	1	1	NA	1	1	1	1	0	0	0	0	0	1
0	1	1	1	1	NA	1	1	1	0	0	0	0	0	1	1
0	1	1	1	1	NA	1	1	1	1	0	0	0	0	0	1
0	1	1	1	1	NA	1	1	1	1	0	0	0	NA	NA	NA

0	1	1	1	1	NA	1	1	1	0	0	0	0	NA	NA	NA
0	1	1	1	1	NA	1	1	1	0	0	0	0	NA	NA	NA
0	1	1	1	1	NA	1	1	1	1	0	0	0	NA	NA	NA
0	1	1	1	1	NA	1	1	1	0	0	0	0	0	0	1
0	1	1	1	1	NA	1	1	1	0	0	0	0	0	0	1
0	1	0	0	0	NA	1	1	0	1	0	0	0	0	0	1
0	1	0	0	1	NA	1	1	1	0	0	0	0	NA	NA	NA
0	1	0	0	1	NA	1	1	1	0	0	0	0	NA	NA	NA
0	1	1	1	1	NA	1	1	1	1	0	1	1	1	1	1
0	1	1	0	0	NA	1	1	1	0	0	0	0	0	0	1
0	1	1	1	1	NA	1	1	1	0	0	0	0	NA	NA	NA
1	1	1	1	1	NA	1	1	NA	1	0	0	0	0	1	1
0	1	1	0	1	NA	1	NA	NA	1	0	0	0	NA	NA	NA
0	0	0	0	0	NA	1	1	0	0	0	0	0	NA	NA	NA
1	1	1	1	0	NA	1	1	1	0	0	0	0	0	1	1
0	1	0	1	0	NA	1	1	1	0	0	0	0	NA	NA	NA
0	1	1	1	0	NA	1	1	1	0	0	0	0	0	1	1
0	1	1	1	1	NA	1	1	NA	1	0	0	0	NA	NA	NA
0	1	1	1	1	NA	1	1	1	1	0	0	0	NA	NA	NA
0	1	1	1	1	NA	1	1	1	1	0	0	0	NA	NA	NA

PEMAT17	LIX	EQIP1	EQIP2	EQIP3	EQIP4	EQIP5	EQIP6	EQIP7	EQIP8	EQIP9	EQIP10	EQIP11	EQIP12	EQIP13
NA	42	0	1	1	1	1	0,5	NA	0,5	0	1	1	1	0
NA	48	0	0	0	0	1	0,5	0	1	0	1	1	1	0
NA	43	0,5	1	1	1	1	0,5	NA	1	0	0,5	1	0	0
NA	39	0,5	1	1	1	1	0,5	0	1	0	0	0	0	0
NA	40	0	1	1	1	1	0,5	0	1	0	0	1	0	0
NA	44	0	0,5	0,5	0,5	1	0,5	1	1	0	0	1	0	0
NA	47	0,5	0,5	0,5	1	1	0,5	NA	0,5	0	0	1	0	0
NA	43	1	1	1	1	1	1	NA	1	0	0	1	1	0
NA	39	0	1	1	1	1	1	1	0,5	0	1	0	0	0
NA	46	0,5	0,5	0,5	1	1	0,5	1	1	0	0,5	1	1	0
NA	49	1	1	1	1	1	0,5	NA	1	0	1	1	0	0
NA	47	0,5	1	1	1	1	0,5	NA	1	0	0,5	1	0,5	0
NA	44	0,5	0,5	0,5	0,5	1	1	0,5	0,5	0	0	1	0	0
NA	37	0,5	1	1	1	1	0,5	NA	1	0	1	1	1	0
NA	50	0,5	1	1	1	1	0,5	NA	1	0	1	1	0	0
NA	43	0	0,5	0,5	0	1	0	1	1	0	0	1	0	0
NA	41	0,5	1	1	1	1	1	NA	1	0	0,5	1	0	0
NA	36	0,5	1	1	1	1	0,5	0,5	0	0	1	0	0	0
NA	44	0	1	1	0,5	1	0,5	0,5	1	0	1	1	0	0
1	43	0,5	1	0,5	0	1	0,5	NA	0,5	0	1	1	1	0
NA	43	0,5	0,5	0,5	0,5	1	0,5	0,5	0,5	0	1	1	1	0
NA	41	0,5	0,5	1	0	1	0,5	0,5	1	0	0	1	0,5	0
NA	41	0	1	1	0,5	1	0,5	NA	0,5	0	0	1	0,5	0
NA	38	0	0,5	1	0	1	0,5	0,5	0,5	0	1	0	0	0
NA	29	0,5	1	1	0,5	1	0,5	0,5	0	0	1	1	1	0
NA	44	0,5	0,5	1	0	1	0,5	0,5	1	0	1	1	0	0
NA	36	0,5	1	1	1	1	1	1	1	0	1	1	0	0
NA	37	0,5	1	1	1	1	0,5	NA	0,5	0	1	1	0	0
NA	31	0	1	1	1	1	0,5	NA	1	0	0	0	0,5	0
NA	36	0,5	1	1	1	1	1	1	1	0	1	0	0	0
NA	40	0,5	1	0,5	1	1	0	0,5	0	0	1	1	1	0
NA	46	0,5	0,5	1	1	1	0,5	0	0,5	0	0,5	1	1	0
NA	35	0,5	0	1	0,5	1	0,5	0,5	0,5	0	1	1	1	0
NA	40	0	1	1	1	1	1	NA	1	0	1	1	0	0
NA	46	0,5	0,5	1	0	1	0	NA	0,5	0	0	1	0,5	0
NA	33	0,5	0	0,5	0	1	0,5	0,5	0,5	0	0	1	1	0
NA	46	0,5	1	0	1	1	0,5	NA	1	0	1	0,5	0	0
NA	47	0,5	1	0	1	1	1	NA	0,5	0	1	1	0	0
NA	49	0	1	0,5	0,5	1	0,5	NA	0,5	0	1	1	0	0
NA	36	0,5	1	1	1	1	0,5	0	0,5	0	0	1	1	0
NA	43	0,5	0	0,5	0,5	1	1	1	0,5	0	1	1	1	0
NA	42	0	1	0,5	0	1	0,5	0	0,5	0	0	1	1	0
NA	47	0	0	0,5	0,5	1	0	0	0,5	0	0	1	1	0
0	38	0	0	0,5	0	0,5	0	0	0	0	0,5	1	0	0
NA	45	0,5	1	1	1	1	0,5	0	0,5	0	1	1	0	0
NA	47	0	1	0,5	1	1	0,5	NA	1	0	0	0	0	0
NA	39	0	1	0,5	1	1	0,5	0	0,5	0	0	1	1	0
NA	47	0	1	1	1	1	0,5	NA	0,5	0	1	1	0	0
NA	44	0	1	1	1	1	1	NA	0,5	0	1	0	0	0
NA	35	0	1	1	1	1	0,5	0	0,5	0	1	1	1	0
NA	39	0	0,5	1	1	1	1	NA	0,5	0	1	0	0,5	0
NA	43	0	0,5	0,5	1	1	0,5	0	0	0	1	1	1	0
NA	37	0	1	1	1	1	0,5	0	0,5	0	0,5	0	0	0
NA	45	0	1	1	1	1	0,5	0	0,5	0	0	1	1	0
NA	35	0,5	1	1	1	1	0,5	0	0,5	0	0,5	0	0,5	0
NA	42	0	1	1	1	1	0,5	NA	0,5	0	1	1	0,5	0

NA	37	0	1	1	1	1	0,5	NA	0,5	0	0	0	0	0
NA	33	0,5	1	1	1	1	1	NA	0,5	0	1	1	0,5	0
NA	41	0	1	1	1	1	0,5	NA	1	0	1	0	0	0
NA	41	0	0,5	1	1	1	0,5	0,5	0,5	0	0,5	0	0	0
NA	41	0	1	1	1	1	0,5	0	0	0	0,5	1	0	0
NA	44	0	0	0,5	0	1	0,5	0	1	0	0,5	1	1	0
NA	46	0	0,5	0	1	1	0,5	NA	0,5	0	1	0	0	0
NA	42	0	0,5	0	1	1	0,5	NA	0,5	0	1	0	0	0
NA	39	0	1	1	1	1	1	1	0,5	0	0,5	0	0	0
NA	39	0	0,5	1	0	1	0,5	0	0,5	0	0	0	1	0
NA	39	0	1	1	1	1	1	NA	0,5	0	1	1	1	0
NA	40	0	1	1	1	1	1	0,5	1	0	0	1	0,5	0
NA	49	0	0,5	0	0,5	1	0,5	NA	1	0	0	1	1	0
NA	53	0	0	0,5	0	1	0,5	NA	0	0	0,5	1	0	0
NA	35	0,5	1	1	0	1	0	0	0	0	0	1	1	0
NA	54	0	0	0	0	1	0	NA	0	0	0	0	0	0
NA	42	0	1	0,5	0	1	0	0,5	0,5	0	1	1	1	0
NA	35	0	1	0,5	1	1	0,5	NA	1	0	0,5	0	0	0
NA	39	0,5	1	1	1	1	0,5	NA	0,5	0	0,5	1	0	0
NA	45	0	1	0,5	1	1	0,5	NA	1	0	1	0	0	0
