# Supplementary material for: Readability, understandability and language accessibility of Swedish websites about the coronavirus disease 2019: a cross-sectional study
Source: BMC Med Inform Decis Mak. 2022 May 13;22:131. doi: 10.1186/s12911-022-01873-y (PMC9103604; doi:10.1186/s12911-022-01873-y)
Supplement: Supplementary file 4 — Additional file 4. Quality scores of the top and bottom three websites in regard to the investigated variables. [file 12911_2022_1873_MOESM4_ESM.pdf]

Additional File 4. Quality scores of the top and bottom three websites in regard to the investigated variables (colored cells indicate that the website was represented in more than one of the variables).

Variable	Quality level	Rank	Affiliation	Score	Website ID
LIX	Easiest readability	1	News	29	W 27
		2	Health care	31	W 31
		3	News	33	W 38
	Most difficult readability	74	Government	50	W 16
		75	Government [a]	53	W 74
		76	Government [b]	54	W 76
EQIP	Highest quality	1	News	75 %	W 8
		2	Health care	75 %	W 15
		3	Government [c]	73 %	W 29
	Lowest quality	74	Government [a]	29 %	W 74
		75	Information website [d]	19 %	W 47
		76	Government [b]	8 %	W 76
PEMAT-PU	Most understandable	1	Government	87 %	W 69
		2	Government [c]	80 %	W 29
		3	Pharmacy	80 %	W 9
	Least understandable	74	News	31 %	W 46
		75	Government [a]	17 %	W 74
		76	Information website [d]	12 %	W 47

[a] Same website affiliated with the government ranked low in three variables (LIX, EQIP, and PEMAT-PU)
[b] Same website affiliated with the government ranked low in two variables (LIX and EQIP)
[c] Same website affiliated with the government ranked high in two variables (EQIP and PEMAT-PU)
[d] Same information website ranked low in two variables (EQIP and PEMAT-PU)
